# Full-length transcriptome sequencing and comparative transcriptome analysis of *Eriocheir sinensis* in response to infection by the microsporidian *Hepatospora eriocheir*

**DOI:** 10.3389/fcimb.2022.997574

**Published:** 2022-12-01

**Authors:** Libo Hou, Mengdi Wang, Lei Zhu, Mingxiao Ning, Jingxiu Bi, Jie Du, Xianghui Kong, Wei Gu, Qingguo Meng

**Affiliations:** ^1^ Engineering Lab of Henan Province for Aquatic Animal Disease Control, College of Fisheries, Henan Normal University, Xinxiang, China; ^2^ Institution of Quality Standard and Testing Technology for Agro-product, Shandong Academy of Agricultural Science, Jinan, Shandong, China; ^3^ Animal Husbandry and Veterinary College, Jiangsu Vocational College of Agriculture and Forestry, Jurong, Jiangsu, China; ^4^ Jiangsu Key Laboratory for Aquatic Crustacean Diseases, College of Marine Science and Engineering, Nanjing Normal University, Nanjing, Jiangsu, China

**Keywords:** *Eriocheir sinensis*, third-generation sequencing technology, microsporidian, intestine, hepatopancreatic necrosis disease

## Abstract

As a new generation of high-throughput sequencing technology, PacBio Iso-Seq technology (Iso-Seq) provides a better alternative sequencing method for the acquisition of full-length unigenes. In this study, a total of 22.27 gigabyte (Gb) subread bases and 128,614 non-redundant unigenes (mean length: 2,324 bp) were obtained from six main tissues of *Eriocheir sinensis* including the heart, nerve, intestine, muscle, gills and hepatopancreas. In addition, 74,732 unigenes were mapped to at least one of the following databases: Non-Redundant Protein Sequence Database (NR), Gene Ontology (GO), Kyoto Encyclopaedia of Genes and Genomes (KEGG), KEGG Orthology (KO) and Protein family (Pfam). In addition, 6696 transcription factors (TFs), 28,458 long non-coding RNAs (lncRNAs) and 94,230 mRNA-miRNA pairs were identified. *Hepatospora eriocheir* is the primary pathogen of *E. sinensis* and can cause hepatopancreatic necrosis disease (HPND); the intestine is the main target tissue. Here, we attempted to identify the key genes related to *H. eriocheir* infection in the intestines of *E. sinensis*. By combining Iso-Seq and Illumina RNA-seq analysis, we identified a total of 12,708 differentially expressed unigenes (DEUs; 6,696 upregulated and 6,012 downregulated) in the crab intestine following infection with *H. eriocheir*. Based on the biological analysis of these DEUs, several key processes were identified, including energy metabolism-related pathways, cell apoptosis and innate immune-related pathways. Twelve selected genes from these DEUs were subsequently verified by quantitative real-time PCR (qRT-PCR) analysis. Our findings enhance our understanding of the *E. sinensis* transcriptome and the specific association between *E. sinensis* and *H. eriocheir* infection.

## Introduction

The Chinese mitten crab *Eriocheir sinensis* is an important freshwater species in the Chinese aquaculture industry due to its high commercial value (Chen and Zhang, 2007). Thus far, many approaches have been applied to study the immunity, development and metabolism of these crabs, including investigations of single gene or global gene functionality by high-throughput sequencing analysis (Robalino et al., 2009; Wang et al., 2022). The combination of whole genome sequencing and transcriptome analysis is a highly effective approach with which to perform systematic studies of gene function. However, *E. sinensis* has a large genome with highly repetitive sequences; thus, a high-quality whole genome sequence is not currently available for this species in public databases. For species without a publicly available whole genome sequence, high-throughput RNA sequencing can play an important role in the investigation of gene function. By applying high-throughput next-generation sequencing (NGS), recent studies have been able to characterise the transcriptome of different tissues in *E. sinensis* and identify several key genes (Cui et al., 2013; Huang et al., 2015). Although the application of NGS can yield a high-throughput database, this approach cannot isolate full-length genes due to the short read length associated with this technology. However, as a new generation of high-throughput sequencing technology, PacBio Iso-Seq can overcome the shortcomings of NGS and yield full-length cDNAs. Thus far, only one Iso-Seq dataset is available for crabs (Wan et al., 2019).

A previous study identified a novel microsporidian pathogen that can infect the hepatopancreatic epithelial cells of *E. sinensis* and investigated the morphological characteristics of this pathogen (Wang and Chen, 2007). This microsporidian pathogen was subsequently identified in non-native *E. sinensis* from Europe and named *Hepatospora eriocheir* (Stentiford et al., 2011). Hepatopancreatic necrosis disease (HPND) is a serious disease that has caused epidemics in cultured stocks of *E. sinensis* since 2015 (Ding et al., 2016). Previous studies of *H. eriocheir* were all based on histological and the phylogenetic analysis of 18S rDNA. Very few studies have addressed the molecular pathogenesis of infections in *E. sinensis* caused by *H. eriocheir*. However, several microsporidian genomes, including those of *H. eriocheir*, have been sequenced over recent years; these studies revealed a loss of certain genes responsible for glycolysis and oxidative phosphorylation. These findings revealed that microsporidians, as an endoparasitic fungi, must obtain energy directly from their host (Boakye et al., 2017). A subsequent NGS study identified significant enrichment in the expression levels of genes related to metabolism-related bioprocesses in the hepatopancreas of *E. sinensis* following infection with *H. eriocheir* (Ding et al., 2018). These results suggested that certain metabolic pathways in the host were influenced by *H. eriocheir* to facilitate the survival and infection of the pathogen.

Over recent years, the combined strategy of Illumina RNA-seq and Iso-Seq has been widely used in different species to acquire comprehensive information, identify gene isomers, and reveal functional diversity at the transcriptional level (Dong et al., 2015; Wang et al., 2019; Qiao et al., 2019; He et al., 2019; Luo et al., 2019; Zhang G et al., 2019). However, this new technology has yet to be applied to crustaceans to analyse the molecular pathogenesis of HPND. In the present study, we aimed to identify the key genes related to *H. eriocheir* infection in the intestines of *E. sinensis* by applying a combination of Iso-Seq and Illumina RNA-seq analyses. Our findings will facilitate the identification of pathogenic mechanisms underlying microsporidian infection at the molecular level.

## Materials and methods

### Animal materials and RNA preparation

**Figure 1:**
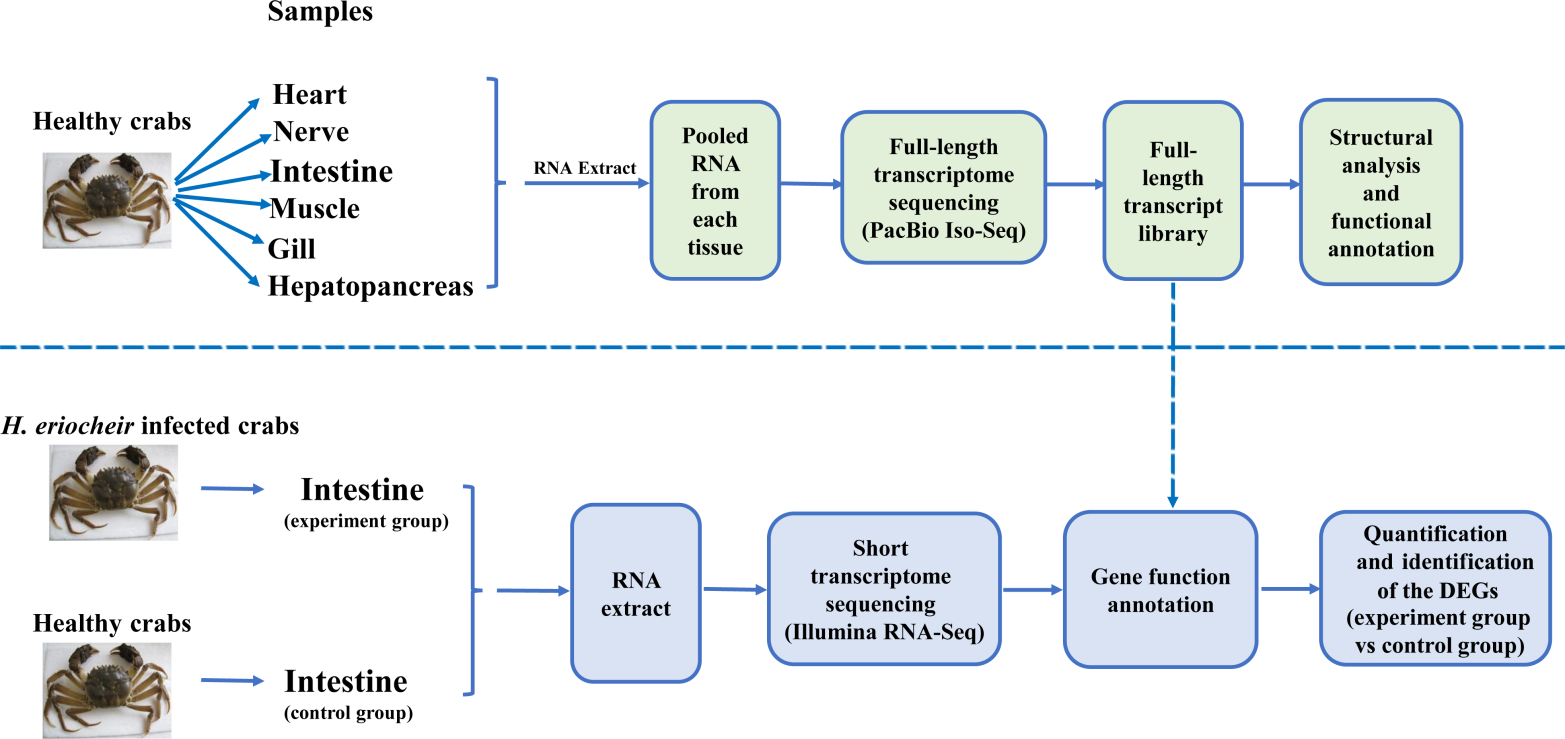
Overview of the experimental design.

For Iso-Seq analysis, a total of six healthy crabs (80 ± 6 g) from Wujin in Jiangsu Province, China were collected at random. Then, we harvested six tissues (heart, nerve (thoracic ganglion), intestine, muscle, gill and hepatopancreas) from each crab. Samples of the same tissue from different crabs were pooled together and immediately stored in liquid nitrogen to await RNA extraction. TRIzol reagent (Thermo Fisher Scientific, USA) was subsequently used to extract total RNA from each pooled tissue. **Figure 1** depicts the overall experimental workflow.

For Illumina RNA-seq analysis, we selected ten *H. eriocheir*-infected crabs (80 ± 6 g) and ten healthy crabs from Wujin in Jiangsu Province, China; then, we harvested the intestines from each crab. All the crabs in the infected (experimental group) had typical symptoms (a white coloured hepatopancreas) and a positive polymerase chain reaction (PCR) result for *H. eriocheir* infection (determined by 16s rDNA sequence analyses). Crabs in the control group did not have any symptoms and were PCR-negative. Intestinal samples were collected and stored in liquid nitrogen for total RNA and DNA purification; three biological replicates were performed. RNA was used for transcriptome analysis while DNA was used to detect and quantify *H. eriocheir* in the intestines of *E. sinensis* by applying methodology described previously by Ding et al. (2017).

### PacBio library and Illumina RNA-Seq library construction

Equal quality of RNA from the six crab tissues were pooled together. Purified polyA (+) RNA (1 μg) was used to synthesize cDNA with the SMARTer PCR cDNA Synthesis Kit (Takara, Japan). Subsequently, the BluePippin Size-Selection System (Sage Science, USA) was used to conduct size fractionation based on a fragment length of 1-2 and >2 kb. Then, we constructed Iso-seq libraries in accordance with the manufacturer’s protocol. P6-C4 reagent was used to sequence the single-molecule real-time (SMRT) cells on the PacBio RS II platform.

In addition, we used poly-T oligo-attached magnetic beads to purify the total mRNA from *H. eriocheir*-positive *E. sinensis* intestines and *H. eriocheir*-negative *E. sinensis* intestines. To obtain high-quality sequencing libraries, we used the NEBNext Ultra RNA Library Prep Kit for Illumina (NEB, USA); library quality was evaluated with an Agilent Bioanalyzer 2100 system.

### PacBio data analysis

The PacBio SMRT Analysis software suite was used to pre-process the raw PacBio data. In brief, after trimming and filtering the raw polymerase reads based on the RS_Subreads protocol, we generated subreads and reads of inserts (ROIs). The filtered conditions were a polymerase read and subread length > 50 bp, polymerase read quality > 0.75 and a minimum of one full pass. Those containing only one 3´-adapter, poly(A) tail and 5´-adapter sequences were regarded as full-length and non-chimeric (FLNC) reads. To obtain high-quality FLNC reads, we used high-quality Illumina short reads (examined by FastQC) to correct errors in the FLNC reads using Proovread software 2.12. Following error correction, the FLNC reads were mapped to IWGSC RefSeq v1.0 using GMAP.

### Functional annotation of genes from Iso-Seq

Next, we performed annotation and extensive gene analysis by applying a range of resources, including euKaryotic Orthologue Groups (KOG), NCBI non-redundant protein sequence (NR), Gene Ontology (GO), Swiss-Prot protein family (Pfam), and Kyoto Encyclopedia of Genes and Genomes (KEGG). The identification of genes coding for conserved TFs was carried out based on the Animal TFDB database compared with the Hmmscan software algorithm (e-value < 0.0001). Next, the CPC2 (http://cpc.cbi.pku.edu.cn/), CPAT (http://lilab.research.bcm.edu/cpat/), PLEK (https://sourceforge.net/projects/plek/), and CNCI (https://github.com/www-bioinfo-org/CNCI) databases were used for lncRNA analysis. Then, we investigated the interactions between mRNAs and miRNAs. For this, we used a crab miRNAs library that we generated previously (Ou et al., 2012) and used online miRanda software (http://www.microrna.org/microrna) to generate mRNA-miRNA interacting networks following common operations and standards (John et al., 2004).

### Gene set enrichment analysis of Iso-Seq data

GO and KEGG analyses were carried out to investigate gene enrichment. The hypergeometric distribution test was used to determine the statistical significance of GO and KEGG enrichments and the Benjamini–Hochberg method was used for multiple-test correction.

### Identification of differentially expressed genes arising from NGS

To identify key genes related to *H. eriocheir* infection in the *E. sinensis* intestine, we compared the trimmed high-quality reads from Illumina sequencing against the full-length reference transcriptome which was generated by PacBio Iso-Seq, a new generation of high-throughput sequencing technology. The differential expression of NGS transcripts was analysed by RNA-Seq by Expectation-Maximization (RESM). Transcript and gene read counts were generated from TPM data by correcting for possible gene length variations across samples that were mainly derived from differential transcript usage; for this, we used the tximport 1.10.0 R package and the “length Scaled TPM” tool. Next, the corrected read count data for each gene was used to estimate expression level in terms of fragments per kilobase of transcript per million fragments mapped (FPKM). Then, the corrected read count data for each gene was imported into the EdgeR tool in the R package to identify differentially expressed genes (DEGs) with a fold change ≥ 2.0, a false discovery rate [FDR]-adjusted *p*-value < 0.05 and an FPKM ≥ 1.

### Gene expression validation

To validate the gene expression data, we first selected 12 DEGs: vascular endothelial growth factor B (*VEGFB*), vascular endothelial growth factor receptor 1 (*VEGFR*), integrin-β1 (*ITGB1*), Integrin-α9 (*ITGA9*), toll-like receptor 1 (*Toll*), relish, p53, ferritin, six-transmembrane epithelial antigen of the prostate (*STEAP3*), MAP1LC3 (*LC3*), autophagy protein 5 (*ATG5*) and Wntless 7 (*WNT7*). Then, we investigated the expression profiles of these genes in the intestines of *E. sinensis* in response to *H. eriocheir* infection by performing qRT-PCR with a SYBR Green Master kit (Roche, Basel, Switzerland); this gene set and method has been described previously (Franzen et al., 2005; Ding et al., 2016; Ding et al., 2018; Li et al., 2019; Bai et al., 2020; Ding, 2021; Li et al., 2021). The primers used for qRT-PCR analysis are given in **Table 1** and were designed based on the crab cDNA library database generated by PacBio Iso-Seq in the current study.

**Table 1:** Primer sequences used in this study.

Gene description	Primer name	Sequence (5′—3′)	Length of amplicon (bp)
Vascular endothelial growth factor B Vascular endothelial growth factor receptor 1 Integrins-β1 Integrins-α9 Toll-like receptor Relish p53 Ferritin Six-transmembrane epithelial antigen of the prostate MAP1LC3 Autophagy protein 5 Wntless 7 18S Ribosomal protein	VEGFB-RT-F VEGFB-RT-R VEGFR1-RT-F VEGFR1-RT-R ITGB1-RT-F ITGB1-RT-R ITGA9-RT-F ITGA9-RT-R Toll-RT-F Toll-RT-R Relish-RT-F Relish-RT-R P53-RT-F P53-RT-R Ferritin-RT-F Ferritin-RT-R STEAP3-RT-F STEAP3-RT-R LC3-RT-F LC3-RT-R ATG5-RT-F ATG5-RT-R WNT7-RT-F WNT7-RT-R 18S-RT-F 18S-RT-R	CAATGTCGAGGAGTGTGGCT CCTCTGCTGCGTCGTATCTT GAGGTCGGAGAGGTTCTTGC GGGTGTCACGTCTTGGTCTT GCAGGGGAACGAAACCTACA AGGTGTTACAGTCGTGCTGG ACGACATTACCCACACCCAC GCTCGTTGGTCCCTTCTTGA TTGGCCTCACTGAACTCACC TGCCTGAGACGCATTACTCC ACAACAGCCGAAGGAGTCAG AGACACTCAGCAGCACTCAC ACCTGTGCCCTTATGTTGCT CTGTCCTTGAAGACTGGCGT ATGTTGTGGAGCGACCAGG GGCAGATGCAATGGTGAGTG TTCCTCTACTGGGGGTGCTT TGATGACTGCGATGAACGGG TAACAGTGGCTTGCTCTGGG TTACAAGATGGTGGAGGCGG CTGGAACCTCACAGCACACT TTATCGTTGCACAGGCCCAT GGTACAGTTCTTCCGGGTGG CACTTGCACTCCGTCCTCAT TCCAGTTCGCAGCTTCTTCTT AACATCTAAGGGCATCACAGA	100 247 137 243 182 112 91 206 79 158 192 182 90

For qRT-PCR analysis, we reverse-transcribed the total RNA into cDNA with a PrimeScript RT reagent Kit (TAKARA, Japan) in accordance with the manufacturer’s instructions. qRT-PCR was then carried out in a 20 µL reaction system (ddH_2_O, 8 µL; cDNA sample, 1 µL (50 ng/µL); primer, 1 µL (0.5 µL of each primer 10 µM); 2×SYBR Premix Ex Taq, 10 µL). qRT-PCR was then carried out using an ABI Quant studio 6 Flex system with a standard program: 98°C for 30 s, followed by 40 cycles of 98°C for 5 s and 58°C for 30 s. The relative expression levels of different genes were calculated according to the 2^-ΔΔCT^ method (Livak and Schmittgen, 2001). Three technical replicates were included for each gene. One-way analysis of variance (ANOVA) and *post hoc* Duncan multiple range tests were used to statistically analyse changes in the expression levels of the selected genes. Significance was set at *P* < 0.05 and 18S was used as an internal control. Pearson correlation analysis was used to compare the similarity of data arising from qRT-PCR and RNA-Seq.

## Results

### Generation of the crab transcriptome by PacBio Iso-Seq

Based on the PacBio Sequel platform, the full-length transcriptome of *E. sinensis* was generated from six tissues: heart, nerve (thoracic ganglion), intestine, muscle, gill and hepatopancreas. A total of 22.27 Gb subread bases and 12,202,043 subreads were obtained by two SMRT cells from the PacBio library. After pre-processing, 527,286 circular consensus sequence (CCS) reads were generated with a mean length of 2,188 bp and a mean read score of 0.99 (**Table 2**). All ROIs were further classified into 482,109 FLNC reads with a mean length of 1,994 bp. After removing redundancies using the CD-Hit program, 266,498 isoforms were obtained with an N50 (defined as the sequence length of the shortest contig at 50% of the total assembly length) of 2,294 bp and 128,614 unigenes with an N50 of 2,634 bp (**Table 3**; **Table S1**).

**Table 2:** PacBio Iso-seq output statistics in *E. sinensis*.

Species	SMRT^1^ cells	Total base (bp)	Polymerase reads	CCS^2^ reads	CCS mean length	Mean read score	Mean passes	5’	3’	Poly-A	Full-Length	FLNC^3^ reads	Mean FLNC length
*E. sinensis*	2	22,266,815,826	12,202,043	527, 286	2,188	0.99	15	519,418	520,909	510,164	502,826	482,109	1,994

^1^SMRT, single-molecule real-time.

^2^CCS, circular consensus sequencing.

^3^FLNC, full-length and nonchimeric.

**Table 3:** Summary for the transcriptome of *E. sinensis* using PacBio Iso-Seq and Illumina RNA-seq.

	PacBio Iso-Seq		Illumina RNA-seq	
Isoforms	Unigenes	Transcripts	Unigenes
Total bases	544,458,894	298,917,638	62,019,645	39,953,571
Total number	266,498	128,614	46,595	34,772
Average length	2,043	2,324	1,331	1,149
Maximum length	11,677	11,677	18,266	18,266
Minimum length	83	83	201	201
N50	2,294	2,634	2,160	1,865

### Efficient gene annotation of full length transcripts

In total, 128,614 non-redundant unigenes were annotated in the public database, including 74,387 (57.84%) in NR, 27,679 (21.52%) in GO, 44,506 (34.60%) in KEGG, 36,337 (28.25%) in KOG and 58,054 (45.14%) in Pfam. A total of 53,882 (41.89%) unigenes were not annotated in these public databases. The statistical results arising from full-length unigene annotations are given in **Table 4**.

**Table 4:** Statistics of unigenes annotation with different databases.

Database	PacBio Iso-Seq		Illumina RNA-seq	
	NO. unigenes annotated	Annotated rate (%)	NO. unigenes annotated	Annotated rate (%)
NR	74,387	57.84%	9,429	27.12%
GO	27,679	21.52%	5,176	14.89%
KEGG	44,506	34.60%	12,947	37.23%
KOG	36,337	28.25%	4,092	11.77%
Pfam	58,054	45.14%	10,128	29.13%

**Figure 2:**
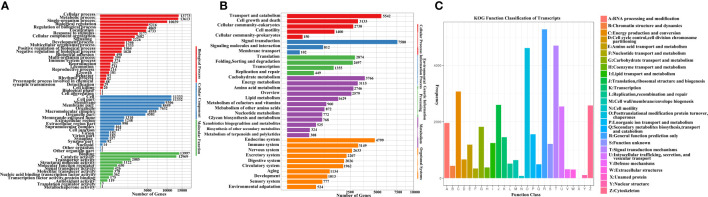
**(A)** Gene Ontology (GO) annotation results for *E. sinensis* full length transcripts; **(B)** Kyoto Encyclopaedia of Genes and Genomes (KEGG) annotation results for *E. sinensis* full length transcripts; **(C)** Karyotic Ortholog Groups (KOG) functional classification of *E. sinensis* full length transcripts.

A total of 27,679 transcripts were mapped to the GO database. GO analysis showed that 20,267 transcripts (73.22%) were assigned to biological processes, 16,935 transcripts (61.18%) were assigned to cellular components, and 22,193 transcripts (80.18%) were assigned to molecular functions. When considering the biological processes, the three most abundant terms were metabolic (49.20%), single-organism (36.27%) and cellular (49.76%) processes. When considering the cellular component category, “cell” and “cell part” were the most abundant terms, accounting for 40.94% of the GO-annotated transcripts. When considering the molecular function category, binding (50.57%) and catalytic activity (46.86%) were the top two subcategories (**Figure 2A**).

A total of 44,506 transcripts were mapped to the KEGG Orthology (KO) database, and 289 signalling pathways were mapped. Based on the number of annotated transcripts, the mapped pathways were classified into five level-1 KO terms. Analysis showed that signal transduction (17.03%), transport and catabolism (12.45%), endocrine (10.78%), and carbohydrate metabolism (8.46%) were the most abundant level-2 KO terms (**Figure 2B**; **Table S2**).

The 36,337 transcripts were grouped into 26 KOG classifications (**Figure 2C**). General functional prediction (5,284, 14.54%) was the largest category, followed by signal transduction mechanisms (4,705, 12.95%), post-translational modifications (4,619, 12.71%), energy production and conversion (3,074, 8.46%), translation, ribosomal structure and biogenesis (2,602, 7.16%), and cytoskeleton (2,581, 7.10%) (**Figure 2C**; **Table S3**).

**Figure 3:**
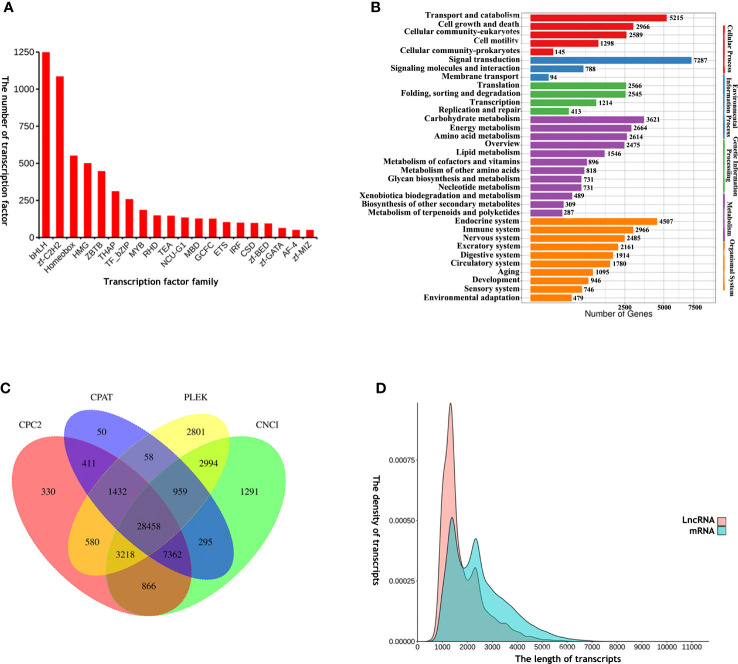
**(A)** The top 20 TFs: basic helix-loop-helix (bHLH), zinc finger C2H2 (zf-C2H2), homeobox, high-mobility-group (HMG), Zinc finger and BTB domain containing (ZBTB), thanatos associated protein (THAP), TF_basic region-leucine zipper (TF_bZIP), v-myb avian myeloblastosis viral oncogene homolog (MYB), Rel homology domain (RHD), TEA domain transcription factor (TEA), Lysosomal transcription factor (NCU-G1), methyl-CpG-binding domain (MBD), GC-rich sequence DNA-binding factor homolog (GCFC), E26 transformation-specific (ETS), interferon regulatory factor (IRF), cold-shock domain (CSD), BED-type zinc finger domain (zf-BED), GATA zinc fingers (zf-GATA), AF4/FMR2 family (AF-4), MIZ-type zinc finger domain (zf-MIZ). The x-axis shows different types of TFs and the y-axis shows the number of TFs; **(B)** KEGG analysis annotation of miRNA-targeted genes obtained, as determined by online miRanda software prediction; **(C)** Venn diagram of lncRNA analysis results; **(D)** Length distribution density analysis of annotated lncRNAs and mRNAs. The x-axis and y-axis represent the length and density of transcripts, respectively.

A total of 6,696 TFs were identified, including basic helix-loop-helix (bHLH), zinc finger C2H2 (zf-C2H2), homeobox, high-mobility-group (HMG), Zinc finger and BTB domain containing (ZBTB) and thanatos associated protein (THAP). THe Animal TFDB 2.0 database classified the identified TFs into 58 families. Of these, bHLH was the most abundant TF (**Figure 3A**; **Table S4**).

A total of 94,230 mRNA-miRNA pairs were identified, including 59,831 mRNAs and 84 miRNAs (**Table S5**). Analysis showed that many mRNAs were targeted by more than one miRNA-based predictive algorithm. KEGG analysis of the targeted genes showed that these genes could be divided into five categories: genetic information processes, metabolism, organismal system, cellular processes, and environmental information processes (**Figure 3B**). These genes are known to be involved in almost all biological processes in cells, including transport and catabolism, signal transduction, energy metabolism and the immune system.

Next, we carried out lncRNA analysis for the *E. sinensis* full-length transcriptome using a customized filtering pipeline. A total of 28,458 lncRNAs were identified in the present study according to coding ability prediction by CPC2, CPAT, PLEK and CNCI (**Figure 3C**; **Table S6**). When compared with mRNAs, the mean length of lncRNAs was relatively short (**Figure 3D**).

### Comparison between FL and *de novo* transcripts

Based on Illumina RNA-seq sequencing, a total of 136.21 million raw reads were obtained from the *E. sinensis* intestine. After processing, 132.81 million clean reads were generated. Based on the obtained clean reads, 46,595 transcripts (mean length 1,331 bp) and 34,772 unigenes (mean length 1,149 bp) were assembled using Trinity software *de novo*. The N50 lengths of the transcripts and unigenes were 2,160 and 1,865 bp, respectively (**Table 3**). Compared with the *de novo* transcripts, the N50 length and mean length of the FL transcripts were longer (**Table 3**).

By searching the public databases NR, GO, KEGG, KOG, and Pfam, a total of 9,429 (27.12%), 5,176 (14.89%), 12,947 (37.23%), 4,092 (11.77%), and 10.128 (29.13%) *de novo* transcripts were obtained, respectively. The most annotated rates from FL transcripts were higher than the transcripts obtained *de novo* (**Table 3**).

### *H. eriocheir* infection and differential expression analysis

Next, we used the FL transcripts obtained in this study as a reference genome sequence and combined these with transcript datasets from the intestines of *E. sinensis* generated by the Illumina sequencing platform to determine the expression values of all 128,614 unigenes in *H. eriocheir*-infected *E. sinensis* intestines (the HE group) and healthy *E. sinensis* intestine (the ES group). Of the 12,708 differentially expressed unigenes (DEUs) with a fold change ≥ 2.0, there were 6,696 upregulated unigenes and 6,012 downregulated unigenes in *H. eriocheir*-infected *E. sinensis* intestines when compared to healthy *E. sinensis* intestines (**Figure S1**; **Table S7**).

**Figure 4:**
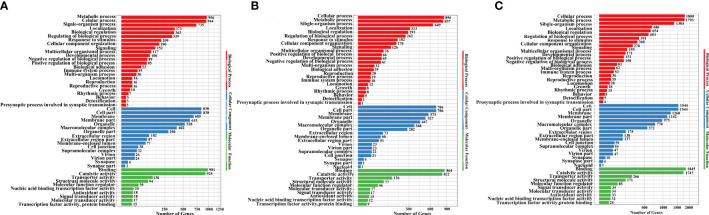
GO enrichment analysis of upregulated unigenes **(A)**, downregulated unigenes **(B)**, and total DEUs **(C)** related to *H. eriocheir* infection in the *E. sinensis* intestine.

Next, we used GO annotation to analyse the DEUs, upregulated unigenes (**Figure 4A**; **Table S8**), downregulated unigenes (**Figure 4B**; **Table S8**) and total DEUs (**Figure 4C**; **Table S8**). Analysis of biological processes showed that the majority of DEUs were associated with cellular, metabolic and signal-organism processes. Analysis of cellular components showed that over half of the DEUs were associated with the cell, cell parts, membranes, and membrane parts. Analysis of molecular function showed that the DEUs were mainly associated with binding, catalytic activity and transporter activity.

**Figure 5:**
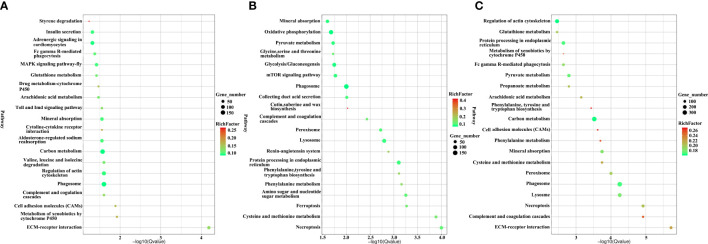
The top 20 signalling pathways of the upregulated unigenes **(A)**, downregulated unigenes **(B)**, and total DEUs **(C)** related to *H. eriocheir* infection in *E. sinensis* intestine based on KEGG enrichment analysis.

KEGG pathway enrichments were used to analyse the biological function of DEUs. A total of 57, 48 and 74 significantly enriched KEGG pathways from upregulated unigenes (**Table S9**), downregulated unigenes (**Table S10**) and total DEUs (**Table S11**) were discovered, respectively, in *H. eriocheir*-infected *E. sinensis* intestines when compared to the intestines of healthy *E. sinensis*. The top 20 different KEGG pathways are shown in **Figures 5A–C**. Of the upregulated unigenes, most of the DEUs were mapped in the following three pathways: metabolism-related pathways (insulin secretion, glutathione metabolism, arachidonic acid metabolism, adrenergic signalling in cardiomyocytes, valine leucine and isoleucine degradation); cytochrome-related pathways (metabolism of xenobiotics by cytochrome P450 and drug metabolism-cytochrome P450); and immune-related pathways (Fc gamma R-mediated phagocytosis, regulation of actin regulation, cell adhesion molecules, cytokine–cytokine receptor interaction, MAPK pathway, ECM-receptor interaction, and the Toll and Imd signalling pathways). When considering the downregulated unigenes, we found that the majority of unigenes were associated with metabolism-related pathways (oxidative phosphorylation, pyruvate metabolism, glycolysis, phenylalanine tyrosine and tryptophan biosynthesis and synaptic vesicle cycles) and immune-related pathways (mTOR signalling pathway, peroxisome, lysosome, ferroptosis, and necroptosis). When considering all DEUs, the majority of unigenes were associated with metabolism-related pathways (glutathione metabolism, pyruvate metabolism, arachidonic acid metabolism, carbon metabolism, oxidative phosphorylation, glycolysis and the pentose phosphate pathway), cytochrome-related pathways (drug metabolism-cytochrome P450 and the metabolism of xenobiotics by cytochrome P450) and immune-related pathways (cell adhesion molecules, ECM-receptor interaction, regulation of actin regulation, Fc gamma R-mediated phagocytosis, phagosome, lysosome, necroptosis, ferroptosis, proteasome peroxisome, Toll and IMD signalling pathway, MAPK pathway and the mTOR signalling pathway).

### qRT-PCR verification of important DEGs

**Figure 6:**
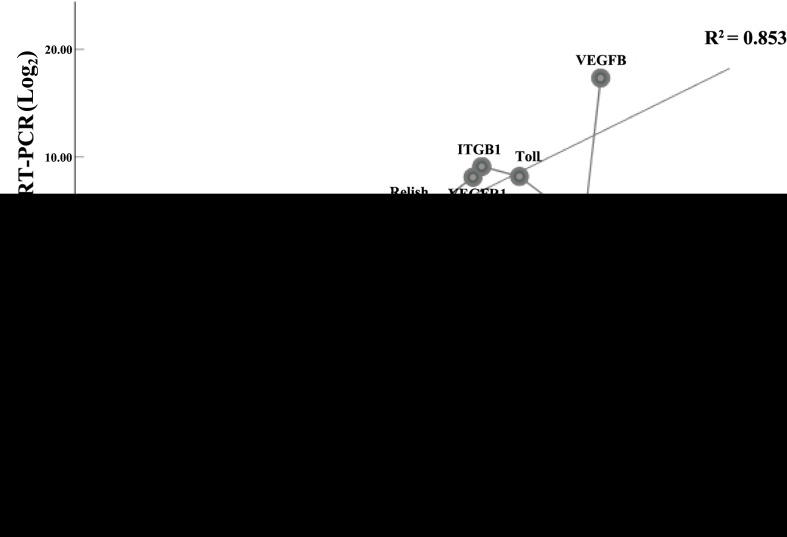
Comparisons of changes in the expression levels of selected DEUs in the intestine as determined by qRT-PCR and by RNA-Seq analysis. Also shown is the relative fold change of target gene expression levels in *H. eriocheir*-infected *E. sinensis* intestine compared to the control group. For qRT-PCR and RNA-Seq comparison, we utilised Pearson’s correlation analysis. The correlation coefficient was 0.924, p-value <0.01. VEGFB: vascular endothelial growth factor B; VEGFR, vascular endothelial growth factor receptor 1; ITGB1, integrins-β1; ITGA9, integrins-α9; Toll, Toll-like receptor 1; Relish; p53; Ferritin; STEAP3, six-transmembrane epithelial antigen of the prostate; LC3, MAP1LC3; ATG5, autophagy protein 5; WNT7, wntless 7.

To further validate data arising from transcriptome analysis, we used qRT-PCR to confirm the changes in expression of 12 selected DEGs. As shown in **Figure 6**, the expression profiles of the 12 selected DEGs were similar to those yielded by RNA-seq (the correlation coefficient was 0.924, *p*-value <0.01). The 12 selected DEGs included six upregulated genes (*VEGFB*, *VEGFR 1*, *ITGB 1*, *ITGA 9*, *Toll* and *Relish*) and six downregulated genes (*P53*, *Ferritin*, *STEAP 3*, *LC3*, *ATG 5* and *WNT7*). We also tested the efficiency of primer amplification.

## Discussion

As an efficient and sensitive means of comprehensive molecular analysis, high-throughput transcriptome sequencing techniques have gradually gained popularity and are now widely used to investigate the molecular biology of crustaceans (Zhong et al., 2017; Li et al., 2018; Hou et al., 2020). Although second-generation high-throughput sequencing has also contributed to crustacean studies, there are still many disadvantages associated with this technology. For example, using such technology, it is difficult to identify alternative splicing (AS), long transcripts and repetitive transposable elements (Michael and VanBuren, 2015; Cheng et al., 2017). With the continuous innovation of sequencing technology, PacBio, as a third-generation sequencing technology, can capture full-length transcripts (containing the 5´ and 3´ ends of cDNAs) without assembly and can overcome the limitations imposed by short-read data. Therefore, third generation sequencing methods have led to significant improvements in research studies targeting species for which there is no published reference genome (Ferrarini et al., 2013). For example, if we want to investigate the functionality of key genes, the first step would be to obtain the full-length cDNA sequences of the target gene. Prior to the advent of third-generation sequencing technology, the lack of a published reference genome meant that the full-length sequence of a target gene could only be obtained by the rapid amplification of cDNA ends (RACE); however, this strategy is labor intensive and expensive (Bower and Johnston, 2010). Therefore, to some degree, third-generation transcriptome sequencing data can avoid the need for a reference genome.

Several studies involving crustaceans have used PacBio to acquire information related to gene expression, including *Litopenaeus vannamei*, *S. paramamosain* and *Exopalaemon carinicauda* (Wan et al., 2019; Zhang X et al., 2019; Zeng et al., 2018; Zeng et al., 2020). These studies showed that the mean length of the PacBio transcripts was over 2 kb, far exceeding the mean lengths of 300 - 1,200 bp obtained by short-read sequencing. In our present study, we used third-generation sequencing technology to generate full-length transcriptomic data from six main tissues of *E. sinensis* with high confidence, including the heart, nerve (thoracic ganglion), intestine, muscle, gill and hepatopancreas. As expected, a total of 22.27 Gb subread bases and 128,614 non-redundant unigenes were obtained with a mean length of 2,324 bp. A total of 74,732 (58.11%) unigenes were annotated in at least one public database (NR, GO, KEGG, KOG, Pfam), far exceeding the annotation rates from short-read sequencing transcripts for *E. sinensis*. In addition, 53,882 (41.89%) unigenes were not annotated in these public databases. This may be due to the absence of a publicly available reference genomic database or because some of these unigenes represent putative novel genes for *E. sinensis*. The large number of full-length cDNA sequences obtained in this study could serve as a basis for the study of full-length transcripts in *E. sinensis*. For example, miRNA, as an important post-transcriptional regulatory molecule, can bind to the 3´-UTR of the target mRNA and thus initiate degradation (Huntzinger and Izaurralde, 2011). Based on the full-length transcripts identified herein, and an *E. sinensis* miRNA library obtained by a previous study (Ou et al., 2012), we were able to generate mRNA-miRNA interaction networks for analysis. These results could serve as a reference for studying the mechanisms of miRNA-related post-transcriptional regulation.

Compared with previous transcriptomic studies of different tissues in *E. sinensis* that utilized NGS (Illumina-Seq), our present data indicated a more extensive transcriptome dataset with notable highlights (Cui et al., 2013; Huang et al., 2015). First, by using the full-length transcript library generated in the present study as a reference database, we were able to improve the gene assembly and annotation of *E. sinensis*. This could help us to better assess the pathogenic mechanisms of microsporidian infection in crab. Second, 128,614 non-redundant full-length unigenes were obtained from *E. sinensis*, thus providing vital data that permits the direct analysis of gene structure and function. *H. eriocheir* is the pathogen responsible for HPND in *E. sinensis*; the intestine of crabs is also the major target tissue of *H. eriocheir* infection. Our goal was to identify the key genes involved in the intestinal infection of *E. sinensis* by *H. eriocheir* by combining PacBio and Illumina RNA-seq technology. In total, 12,708 differentially DEUs (6,696 upregulated and 6,014 downregulated) were identified in the intestines of *H. eriocheir*-infected *E. sinensis* when compared to the intestines of healthy crab. Biological analysis of these DEUs showed that many pathways or processes participated in interactions between the host cell and *H. eriocheir* infection. As a widespread obligate intracellular parasite, microsporidians are formally classified as fungi (Hirt et al., 1999). In eukaryotes, ATP synthesis is mostly dependent on the oxidative phosphorylation and glycolysis pathways. In microsporidians, however, these two fundamental energy generation pathways have largely disappeared; instead, these organisms are believed to obtain their energy directly from host-derived ATP (Boakye et al., 2017). In this study, transcriptomic analysis found that many ATP-binding proteins and/or ATP metabolism-related proteins underwent significant changes in the intestines of *H. eriocheir*-infected *E. sinensis* when compared with the control group, including ATP-binding cassette (ABC) family F member 2/3 (ABCF2/3), ABCE1/2, ABCA3, vacuolar ATP synthase subunit B (V-ATPase B) and V-ATPase A/E/H. These results indicate that *H. eriocheir* may change the expression levels of these key genes to hijack the host’s energy for itself. In addition to ATP-related proteins, many proteins related to the oxidative phosphorylation and glycolysis pathways were shown to undergo significant changes in the *H. eriocheir*-infected *E. sinensis* intestine, including genes encoding glycolysis-related proteins, such as 6-phosphogluconate dehydrogenase (6-PGDH), triosephosphate isomerase (TPI) and enolase, and genes encoding proteins associated with the tricarboxylic acid cycle (TCA cycle), including succinate dehydrogenase (SDH), phosphoenolpyruvate-carboxykinase (PEPCK) and isocitrate dehydrogenase (IDH). These results suggest that *H. eriocheir* infection seriously disrupts energy metabolism in host cells. Except for energy metabolism, GO and KEGG enrichment analyses of the DEUs also found that *H. eriocheir* infection had significant and negative effects on metabolism and nutrition in the *E. sinensis* intestine. For example, *H. eriocheir* infection significantly changed the expression levels of genes involved in “glutathione, arachidonic acid, pyruvate metabolism”, the “pentose phosphate pathway”, “metabolism”, “arachidonic acid metabolism” and “carbon metabolism”. These findings indicated that *H. eriocheir* may lose energy and exert deleterious effects on the energy metabolism of host cells. This is consistent with previous studies showing that *H. eriocheir* can destroy hepatopancreas tissue in *E. sinensis* and exert negative effects on metabolism (Ding et al., 2018).

It has been observed that to enter a host cell, microsporidians exploit receptor-dependent phagocytosis as their primary mechanism of entry (Franzen et al., 2005). As the most important cell surface receptor, the family of integrins is exploited by many pathogens and then induces cytoskeletal rearrangements to initiate phagocytosis in the host cell, ultimately facilitating the entry of pathogens into the host cell (Watarai et al., 1996; Yilmaz et al., 2002). In the current study, the expression levels of many integrin-dependent phagocytosis-related proteins, including integrins, Arp2/3, Rho GAP, Rho GEF, Rab2, Rab11 and Rab21, were significantly upregulated in the *H. eriocheir*-infected *E. sinensis* intestine. These results suggest that *H. eriocheir* might upregulate the expression levels of genes encoding many integrin-dependent and phagocytosis-related proteins to initiate phagocytosis and facilitate entry into host cells. After entering the host cell, pathogens require a relatively stable environment for survival and infection. During the process of evolution, many pathogens have adopted a series of strategies to inhibit host cell apoptosis or programmed cell death to ensure their latency or reproduction (Gao and Abu Kwaik, 2000; Heussler et al., 2001). The mitochondrial cytochrome C (Cyt-C)/Caspase pathway and the p53-dependent pathway are the main apoptosis-regulated pathways (Garrido et al., 2006; Elmore, 2007; Le Pen et al., 2016). In the present study of *H. eriocheir*-infected *E. sinensis* intestines, the expression levels of Cyt-C, Cyt-b1/5, several types of cytochrome c oxidase (COX) and caspase 3 were all significantly downregulated. These results suggest that *H. eriocheir* infection inhibits apoptosis in host cells. Consistent with this result, the expression levels of anti-apoptotic proteins (cytochrome P450-CYP2/4C/3A24/3A19 and Bcl2) were significantly increased. Furthermore, the expression levels of p53 and TNF-α-factor were significantly suppressed in *H. eriocheir*-infected *E. sinensis* intestines when compared with the control group. Based these results, we speculate that *H. eriocheir*, as an obligate intracellular parasite, adopts specific mechanisms to prevent apoptosis in host cells, thus ensuring latency and reproduction in host cells.

KEGG pathway enrichment analysis of the DEUs identified in this study showed that many immune-related pathways were activated in response to *H. eriocheir* infection. Antimicrobial protein peptides (AMPs), the first line of host protection against pathogenic infection, are indispensable components of innate immunity in invertebrates. In the current study, three types of AMPs were significantly upregulated in the *H. eriocheir*-infected *E. sinensis* intestine when compared to healthy *E. sinensis* intestines, including anti-lipopolysaccharide factor (ALF1, ALF2, ALF3 and ALF5), lysozyme (Lyz1 and Lyz2) and crustin2. Previous studies have shown that in invertebrates, many pathways or proteins are closely related to the expression levels of AMPs. The VEGFR/MAPK pathway is one of the most highly conserved signaling pathways directly involved in the regulation of many innate immune genes, especially in terms of regulating the expression levels of AMPs in the host. For example, the p38 MAPK signaling pathway can regulate the intestinal expression of AMP in *Caenorhabditis elegans* and enhance innate immunity in host cells (Troemel et al., 2006). Similarly, the silencing of ERK in hemocytes from *E. sinensis* by RNA interference led to a significant downregulation in the expression levels of AMPs (Li et al., 2019). In the present study, the expression levels of many proteins or kinases were significantly upregulated, including two VEGFR proteins (VEGFR1 and VEGFR2), the VEGFR ligand VEGF, MAPK, MKNK1 and MnK1. These results suggest that the VEGFR-MAPK pathway is one of the most important pathways in the *E. sinensis* intestine and helps to regulate *H. eriocheir* infection to protect host cells against *H. eriocheir* infection. As an evolutionarily conserved pathway, the Toll and IMD immune signaling pathways are the two major immune pathways responsible for regulating the expression of AMP in invertebrates. The overexpression or knockdown of Toll-like receptors in *Procambarus clarkia* and *Macrobrachium rosenbergii* led to significant changes in the expression of AMP (Wang et al., 2015; Lan et al., 2016; Li et al., 2021). In the present study, the expression levels of Toll1 and Toll2 were significantly upregulated in *H. eriocheir*-infected *E. sinensis* intestines. As one of the key members of the nuclear transcription factor (NF-κB) family, Relish can be activated by the IMD signaling pathway to then regulate AMP expression. In *E. sinensis*, the silencing of Relish in crab hemocytes was shown to significantly restrain the expression levels of AMP (Bai et al., 2020). In the present study, the expression levels of Relish were significantly upregulated in *H. eriocheir*-infected *E. sinensis*. These results suggest that the Toll and IMD signaling pathways also play an important role in the host against *H. eriocheir* infection by regulating AMP expression, although the specific mechanisms involved need to be further validated. As an indispensable aspect of the invertebrate innate immune system, the proPO system can be activated by peptidoglycan (PGN) or lipopolysaccharides (LPS) in bacteria and by β-1,3-glucan in fungi (Söderhäll and Cerenius, 1998). Once activated, this system initiates a series of downstream reactions to convert proPO into the active form PO to then kill invading pathogens (Cerenius and Söderhäll, 2004; Amparyup et al., 2012). In a recent study, the expression levels of many genes encoding proteins in the proPO system were shown to undergo significant changes in *H. eriocheir*-infected *E. sinensis* intestines when compared with the control group, including significant increases in the expression of serine protease, prophenoloxidase-activating factor (PPAE), proPO, clotting protein precursor and clottable protein; in contrast, there were significant reductions in the expression levels of the Kazal-type protease inhibitor. These results indicated that the proPO system, as a major immune response in a host against *H. eriocheir* infection, was activated in *H. eriocheir*-infected *E. sinensis* intestines. Although certain biological processes and innate immune pathways (for example, the proPO system, VEGF/MAPK pathway, Toll and IMD pathway) were found to be involved in the response of *E. sinensis* against *H. eriocheir* infection in the present study, further investigations are now needed to verify how these key pathways participate in protecting crab against *H. eriocheir* infection.

## Conclusion

**Figure 7:**
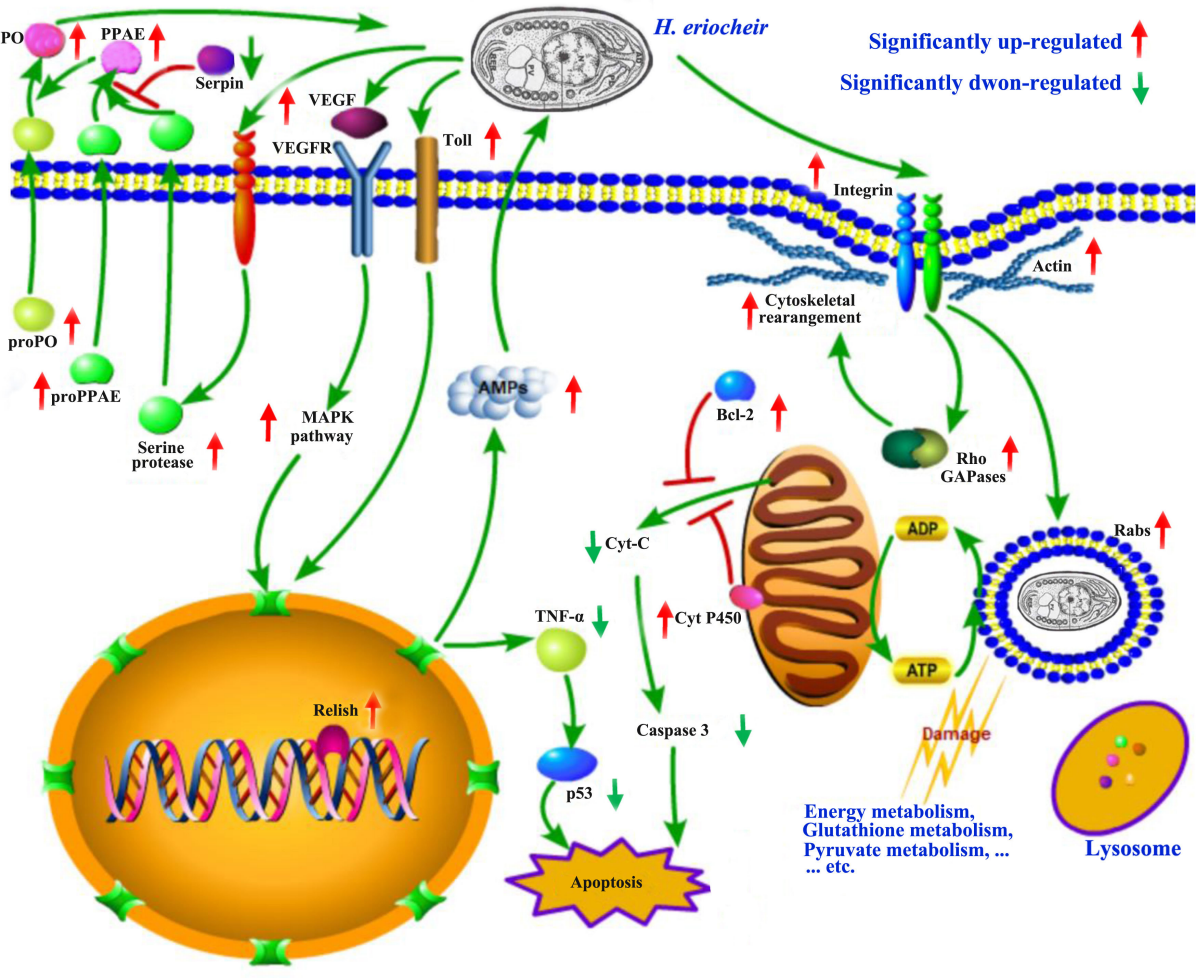
A schematic model of the major biological processes and pathways associated with infection of the *E. sinensis* intestine by *H. eriocheir* (generated by the Pathway Builder Tool). For abbreviations and explanations, see the text.

For the first time, we generated and sequenced a full-length cDNA library consisting of multiple *E. sinensis* tissues (heart, nerve (thoracic ganglion), intestine, muscle, gill and hepatopancreas) by applying PacBio sequencing. This is also the first study to combine PacBio and Illumina RNA-seq analysis in the *E. sinensis* intestine to identify the key genes involved in *H. eriocheir* infection. In **Figure 7**, we present a theoretical mechanism of how *H. eriocheir* infects *E. sinensis*. To survive inside host cells, *H. eriocheir* first exploits integrin-dependent phagocytosis to enter the host cell. Next, *H. eriocheir* deprives energy and exerts detrimental effects on the energy metabolism in the host cells, thus resulting in abnormal metabolism. Therefore, *H. eriocheir* adopts certain mechanisms to prevent apoptosis in host cells to ensure latency and reproduction. In addition, several important innate immune responses are activated in the host cells to protect against infection by *H. eriocheir*. These responses include activation of the MAPK pathway and the Toll and IMD signalling pathways. There is also an increase in the expression levels of AMP in host cells; this activates the proPO system which targets and kills invading *H. eriocheir*. Our findings enhance our understanding of the interaction between *E. sinensis* and *H. eriocheir*.

## Data availability statement

The datasets presented in this study can be found in online repositories. The names of the repository/repositories and accession number(s) can be found in the article/**Supplementary Material**.

## Author contributions

QM, XK, WG, and LH conceived and designed the experiments. LH, MW, LZ, JB, MN, and JD performed the experiments and analyzed the data. LH and QM wrote and edited the draft manuscript. QM was responsible for forming the hypothesis; project development; data coordination, finalizing, and submitting the manuscript. All authors contributed to the article and approved the submitted version.

## Funding

This research was supported by grants from the Jiangsu Agriculture Science and Technology Innovation Fund (No. CX (21) 3157), the Modern Agricultural Industry Technology System Project of Jiangsu Province (Grant No. JATS [20210] 340), National Natural Sciences Foundation of China (NSFC No. 32202985), the Agricultural Scientific and Technological Innovation Project of Shan-dong Academy of Agricultural Sciences (18200214442027, 18200214442026) and the Youth Support Project of Jiangsu Vocational College of Agriculture and Forestry (No. 2020KJ011). The raw data arising from transcriptome sequencing in this study have been deposited in the NCBI BioProjects database with the accession number PRJNA858988.

## Conflict of interest

The authors declare that the research was conducted in the absence of any commercial or financial relationships that could be construed as a potential conflict of interest.

## Publisher’s note

All claims expressed in this article are solely those of the authors and do not necessarily represent those of their affiliated organizations, or those of the publisher, the editors and the reviewers. Any product that may be evaluated in this article, or claim that may be made by its manufacturer, is not guaranteed or endorsed by the publisher.

## Footnotes

1 SMRT: single-molecule real-time;

2 CCS: circular consensus sequenceing;

3 FLNC: full-length and nonchimeric;

## Supplementary material

Supplementary Figure 1 MA plot (M-versus-A plot) and volcano plot for the FL transcripts.

Supplementary Table 1 The characteristics of unigenes from the full-length transcriptome.

Supplementary Table 2 The KEGG classification of all unigenes.

Supplementary Table 3 The KOG classification of all unigenes.

Supplementary Table 4 Transcription factors analysis of all unigenes.

Supplementary Table 5 miRNA-mRNA interaction analysis.

Supplementary Table 6 LncRNA analysis of all unigenes.

Supplementary Table 7 All DEUs related to *H. eriocheir*-infected *E. sinensis* intestine.

Supplementary Table 8 GO enrichment analysis of different expressed unigenes in the intestines of *E. sinensis* following *H. eriocheir* infection.

Supplementary Table 9 KEGG enrichment analysis of unigenes showing upregulated expression in *E. sinensis* intestine after *H. eriocheir* infection.

Supplementary Table 10 KEGG enrichment analysis of unigenes showing downregulated expression in *E. sinensis* intestine after *H. eriocheir* infection.

Supplementary Table 11 KEGG enrichment analysis of all DEUs in *E. sinensis* intestine after *H. eriocheir* infection.
