# Supplementary figures and images for: Full-length transcriptome sequencing and comparative transcriptome analysis of Eriocheir sinensis in response to infection by the microsporidian Hepatospora eriocheir
Source: Front Cell Infect Microbiol. 2022 Dec 1;12:997574. doi: 10.3389/fcimb.2022.997574 (PMC9754153; doi:10.3389/fcimb.2022.997574)

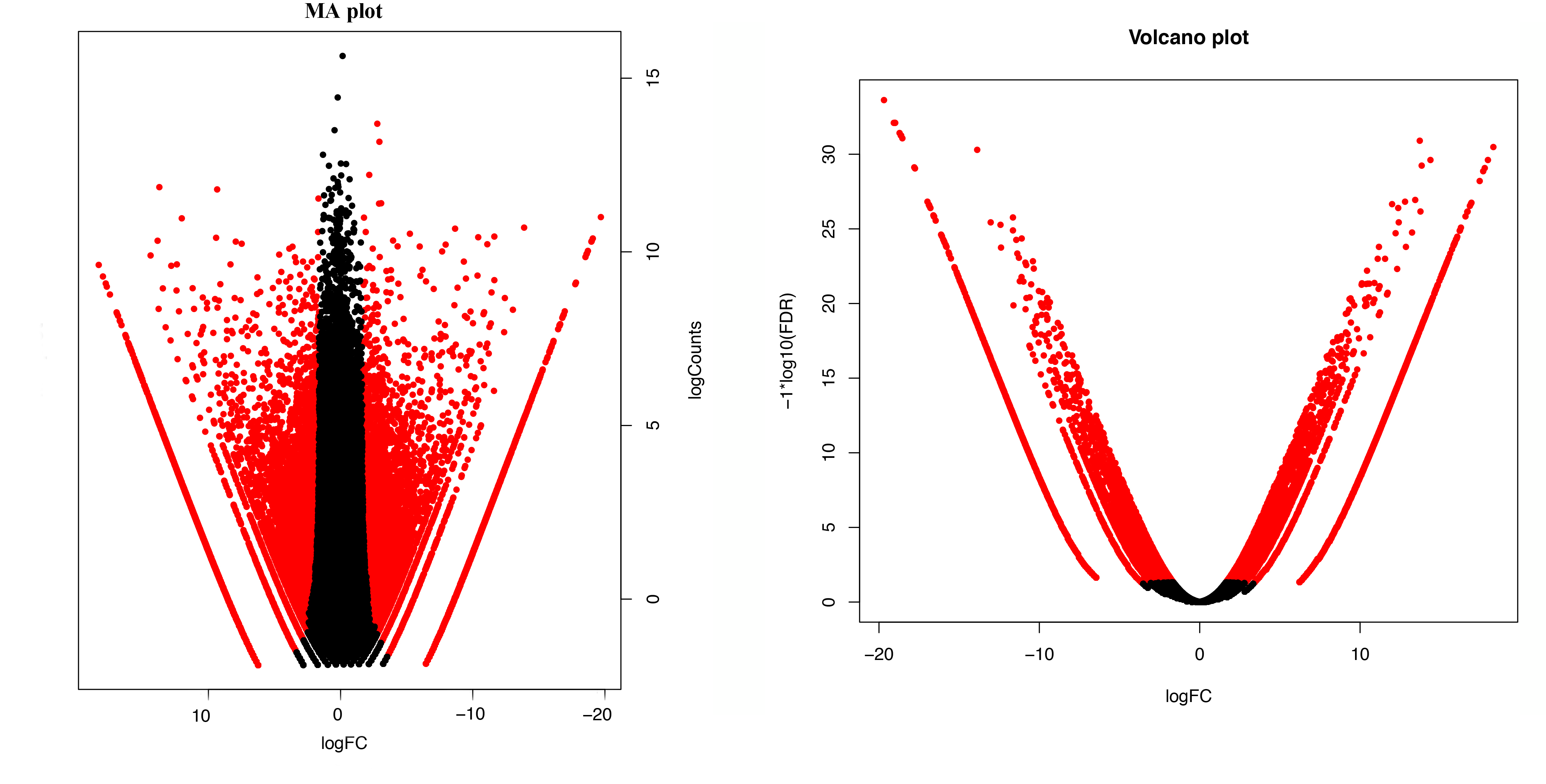

Supplement: Supplementary Figure 1 — MA plot (M-versus-A plot) and volcano plot for the FL transcripts. [file Image_1.tif]
